# Impaired myocardial perfusion is associated with increasing end-systolic- and end-diastolic volumes in patients with non-ischemic systolic heart failure: a cross-sectional study using Rubidium-82 PET/CT

**DOI:** 10.1186/s12872-019-1047-x

**Published:** 2019-03-22

**Authors:** Christina Byrne, Philip Hasbak, Andreas Kjaer, Jens Jakob Thune, Lars Køber

**Affiliations:** 1 Department of Cardiology, The Heart Centre, Rigshospitalet, University of Copenhagen, 9441, Blegdamsvej 9, 2100-Cph Copenhagen, Denmark; 2 Department of Clinical Physiology, Nuclear Medicine & PET and Cluster for Molecular Imaging, Rigshospitalet and University of Copenhagen, Copenhagen, Denmark; 3 Department of Biomedical Sciences, Rigshospitalet and University of Copenhagen, Copenhagen, Denmark; 4 Faculty of Health Sciences, University of Copenhagen, Copenhagen, Denmark; 5 Department of Cardiology, Bispebjerg Hospital, University of Copenhagen, Copenhagen, Denmark

**Keywords:** Myocardial perfusion imaging, Myocardial flow reserve, Non-ischemic systolic heart failure, End systolic volume, End diastolic volume

## Abstract

**Background:**

Myocardial flow reserve (MFR, stress/rest myocardial blood flow) is a strong marker of myocardial vasomotor function. MFR is a predictor of adverse cardiac events in patients with non-ischemic systolic heart failure and previous studies using different methods have found association between myocardial blood flow and left ventricular dilatation. The aim of this study was to investigate whether there is an association between increasing end-systolic- and end-diastolic volumes (ESV and EDV) and MFR in these patients measured with Rubidium-82 positron emission tomography computed tomography (^82^Rb-PET/CT) as a quantitative myocardial perfusion gold-standard.

**Methods:**

We scanned 151 patients with non-ischemic heart failure with initial left ventricular ejection fraction ≤35% with ^82^Rb-PET/CT at rest and adenosine-induced stress to obtain MFR and volumes. To account for differences in body surface area (BSA), we used indexed ESV (ESVI): ESV/BSA (ml/m^2^) and EDV (EDVI). We identified factors associated with MFR using multiple regression analyses.

**Results:**

Median age was 62 years (55–69 years) and 31% were women. Mean MFR was 2.38 (2.24–2.52). MFR decreased significantly with both increasing ESVI (estimate − 3.7%/10 ml/m^2^; 95% confidence interval [CI] -5.6 to − 1.8; *P* < 0.001) and increasing EDVI (estimate − 3.5%/10 ml/m^2^; 95% CI -5.3 to − 1.6; *P* < 0.001). Results remained significant after multivariable adjustment. Additionally, coronary vascular resistance during stress increased significantly with increasing ESVI (estimate: 3.1 mmHg/(ml/g/min) per (10 ml/m^2^); 95% CI 2.0 to 4.3; *r* = 0.41; *P* < 0.0001) and increasing EDVI (estimate: 2.7 mmHg/(ml/g/min) per (10 ml/m^2^); 95% CI 1.6 to 3.8; *r* = 0.37; *P* < 0.0001).

**Conclusions:**

Impaired MFR assessed by ^82^Rb-PET/CT was significantly associated with linear increases in ESVI and EDVI in patients with non-ischemic systolic heart failure. Our findings support that impaired microvascular function may play a role in heart failure development. Clinical trials investigating MFR with regard to treatment responses may elucidate the clinical use of MFR in patients with non-ischemic systolic heart failure.

**Trial registration:**

Sub study of the randomized clinical trial: A DANish randomized, controlled, multicenter study to assess the efficacy of Implantable cardioverter defibrillator in patients with non-ischemic Systolic Heart failure on mortality (DANISH), ClinicalTrials.gov Identifier: NCT00541268.

**Electronic supplementary material:**

The online version of this article (10.1186/s12872-019-1047-x) contains supplementary material, which is available to authorized users.

## Background

Myocardial flow reserve (MFR, stress/rest myocardial blood flow) is a strong marker of myocardial vasomotor function and quantification of MFR may identify microvascular dysfunction [1–3]. MFR is defined as the maximal myocardial blood flow (MBF) during pharmacologic stress divided by resting MBF. In patients with non-ischemic systolic heart failure, MFR is often impaired despite the absence of coronary artery disease [4–6]. Different explanations have been suggested, such as endothelial dysfunction and impaired angiogenesis, which may lead to small vessel disease with impaired perfusion and myocardial ischemia [7]. However, the mechanism behind the impaired perfusion remains unclear.

A detailed evaluation of the underlying pathophysiology of heart failure is important for development and application of new therapies [8]. For detection of microvascular disease without structural epicardial coronary artery disease, ^82^Rb-PET/CT with adenosine stress is a robust and validated method [9, 10]. Outcome studies have shown that MFR is a predictor of adverse cardiac events in patients with non-ischemic systolic heart failure [4, 5]. Moreover, it is well known that severity in left ventricular dysfunction with increased end-systolic volume (ESV) and end-diastolic volume (EDV) at the time of referral is a prognostic indicator of mortality [11].

Previous studies using echocardiography with coronary Doppler catheter or single photon emission computed tomography (SPECT) in patients with non-ischemic cardiomyopathy have shown that myocardial perfusion is negatively correlated with ESV and EDV [12–14]. PET is considered the gold standard of myocardial perfusion measurement. To our knowledge the association between myocardial perfusion, measured quantitatively as MFR, and ESV and EDV using ^82^Rb-PET/CT has not previously been investigated in patients with non-ischemic systolic heart failure of varying etiology. Therefore, the aim of our study was to investigate whether MFR was associated with increasing ESV and EDV in patients with non-ischemic systolic heart failure assessed with ^82^Rb-PET/CT.

## Methods

### Study population

**Fig. 1:**
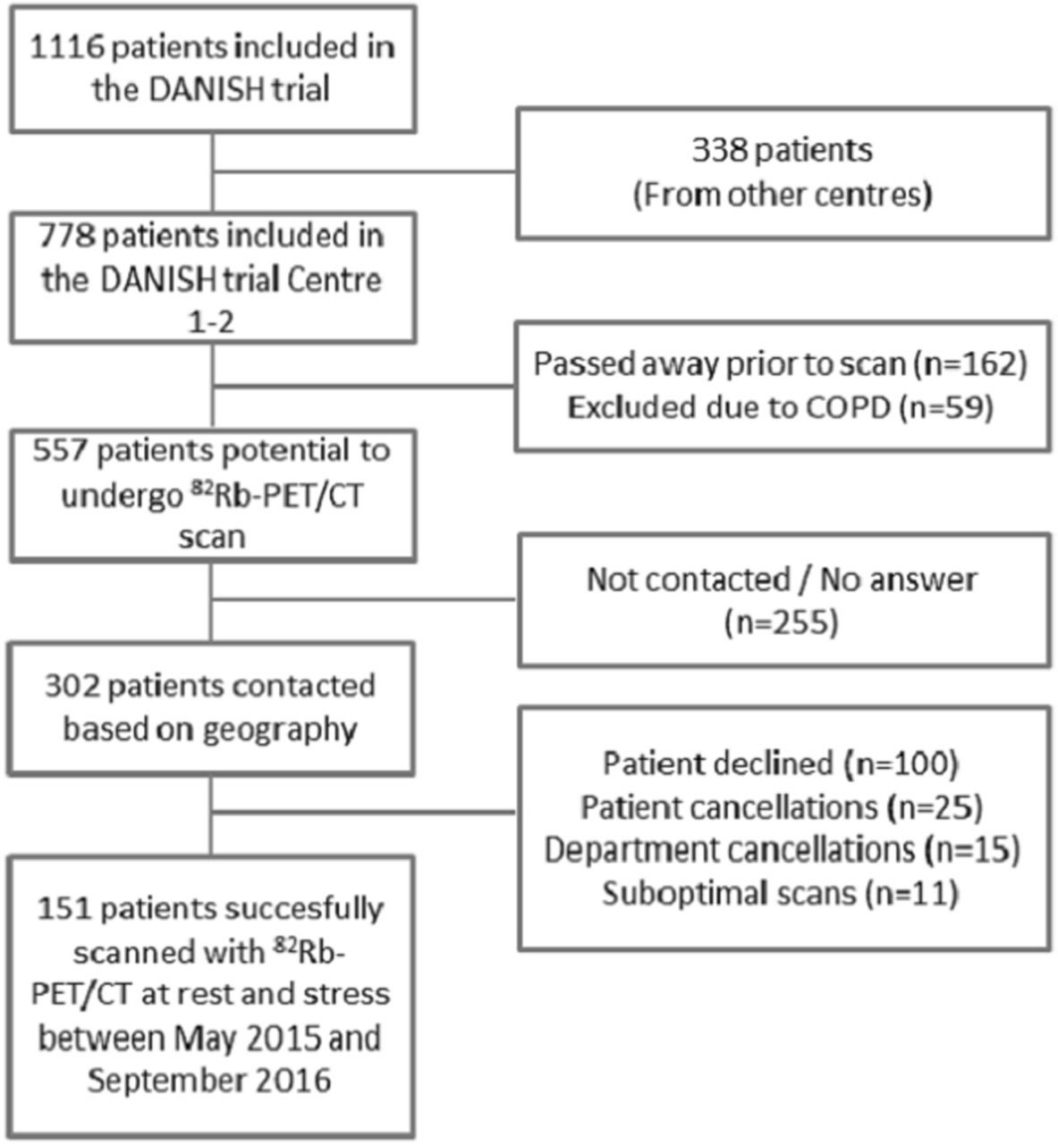
Inclusion flow chart. DANISH [15] Centre 1 and 2: Rigshospitalet and Gentofte Hospital, Copenhagen, Denmark. COPD chronic obstructive pulmonary disease

In this sub study to DANISH (A DANish randomized, controlled, multicenter study to assess the efficacy of Implantable cardioverter defibrillator in patients with non-ischemic Systolic Heart failure on mortality) [15], patients were included late in the follow up period of the main trial. Inclusion criteria for DANISH were documented non-ischemic systolic heart failure (LV ejection fraction ≤35%), and increased levels (> 200 pg/mL) of N-terminal pro-brain natriuretic peptide (NT-proBNP) regardless of optimal medical treatment. Coronary artery catheterization was performed in 97.4% of patients in this sub study to exclude ischemia as the cause of heart failure and CT angiograms was performed in the remaining patients. If an extent of coronary artery disease was not considered to be sufficient to account for the reduced left ventricular systolic function, patients with one or two coronary arteries with stenoses could be included [15]. We excluded patients with severe chronic obstructive pulmonary disease (COPD)/asthma, blood pressure > 200/110 mmHg or systolic blood pressure < 90 mmHg, allergy or intolerance to theophylline or adenosine, pregnancy and inability to adhere to the protocol. Between May 2015 and September 2016, we scanned 151 patients with non-ischemic systolic heart failure with ^82^Rb-PET/CT. Figure 1 shows the inclusion flowchart. All patients had given informed oral and written consents, and the Scientific Ethics Committee of the Capital Region of Denmark and the Danish Data Protection Agency approved the protocol. (protocol number H-15000346). Our study was performed in accordance with the principles of the Declaration of Helsinki.

### PET imaging

All patients underwent rest and adenosine stress PET myocardial perfusion imaging (MPI) during one scan session. Patients were instructed to abstain from caffeine and theophylline for 12 h before acquisition of the scan. Medications prescribed for heart failure were continued by all patients. Patients were scanned with a Siemens Biograph mCT/PET 128-slice scanner (Siemens Healthcare, Knoxville, TN, USA), and rest and stress images were obtained ECG-gated in list mode for 7 min from the start point of ^82^Rb infusion. For rest and stress imaging, patients received 1062 MBq (IQR: 1018-1273) and 1058 MBq (IQR: 1014-1269) ^82^Rb, supplied from a CardioGen-^82^Sr/^82^Rb generator manufactured for Bracco Diagnostics Inc., Princeton, NJ. For the stress scan we used adenosine infusion (0.14 mg/kg/min) as stressor for 6 min and we initiated the stress ^82^Rb infusion 2.5 min after starting the adenosine administration. Also, we performed an attenuation correcting low-dose CT before the rest study, and if necessary repeated it after the stress study. As per clinical routine, coronary artery calcium score (CACS) images from a non-contrast breath-hold CT was acquired in all patients. We calculated the CACS using the Agatston score system [16].

### Quantitative and semi quantitative analyses

For quantification of MBF of dynamic rest and stress images, we used the method previous described in details by Armstrong et al. [17] Based on a single-compartment model for ^82^Rb tracer kinetics suggested by Lortie et al. [10], we conducted the MBF quantification with QGS/QPS v. 2015.5 (Cedars-Sinai Cardiac Suite, Los Angeles, USA). MFR was defined as MBF during adenosine induced maximal hyperemia divided by resting MBF. In order to correct the resting MBF for baseline work, we calculated the rate pressure product (RPP), defined as the systolic blood pressure times the heart rate, and divided MBF with RPP and multiplied by 10,000 [18]. Moreover, we divided MFR into normal (> 2.5), borderline (> 2.0–2.5), and low (≤2.0) [3]. Definitions of resting and stress MBF normal values were 0.82 ml/g/min ±30% and 3.3 ml/g/min ±31%, respectively [19]. Additionally, the coronary vascular resistance (CVR) was calculated as mean arterial pressure (MAP) divided by MBF at rest and during stress. Segmental perfusion scores based on a 17-segment, multi-point scale with corresponding summed scores were automatically calculated as summed rest score (SRS), summed stress score (SSS) and summed difference score (SDS=SSS-SRS) with the QPET software from Cedars-Sinai, using a 17 myocardial segment model [20]. Definition of SSS was: 0–3: normal (< 5% myocardium with perfusion abnormalities), 4–7: mildly abnormal (5–10% myocardium with perfusion abnormalities), > 8 moderately or severely abnormal (> 10% myocardium with perfusion abnormalities) [21]. Left ventricular ejection fraction (LVEF), systolic and diastolic volume measures were also computed by the software and manual corrections were made if the automatic data processing algorithm was unable to generate an accurate LV contour. Normal values for ESV and EDV at rest has been defined in previous studies by Bravo et al. (mean rest ESV ± SD: 42 ± 17 ml and mean rest EDV ± SD: 82 ± 26 ml (15% men, mean age ± SD 49 ± 9 years)) [22] and Chander et al. (mean rest ESV ± SD: 47 ± 31 ml and mean rest EDV ± SD: 81 ± 34 ml (54% men, mean age ± SD 54 ± 12 years)) [23]. To account for differences in body surface area (BSA), we used indexed ESV (ESVI): ESV/BSA (ml/m^2^) and EDV (EDVI): EDV/BSA (ml/m^2^).

### Statistical analyses

Continuous variables were expressed as medians and interquartile ranges. For analyses of differences between groups we used chi-square test for categorical variables. For continuous variables, we used unpaired t-test or Wilcoxon two-sample test. When appropriate, to approximate a normal distribution, variables were log-transformed. To be able to compare our results with previously described normal values, we also calculated the mean ESV and EDV in addition to median values even though not perfectly normal distributed. Linear relations were tested for more complex models of fit using the Akaike information criterion. Multiple regression analysis of explanatory variables (sex, age, hypertension, diabetes, NT-pro-BNP, LV bundle branch block, LV ejection fraction, atrial fibrillation during scan, increases in heart rate from rest to stress and coronary calcium score) was performed with the general linear model (GLM) procedure. *P*-values < 0.05 were considered statistically significant. Statistical analyses were performed with SAS version 9.4 (SAS Institute, Cary, NC, USA). GraphPad Prism 7.02® (GraphPad Software Inc., USA) was used for graphic presentation of results.

## Results

Comparing the 151 patients in our study with the study population of the main study without COPD, we only found few differences (Table 1). Patients in our sub study had lower NT-proBNP (851 vs. 1220 pg/ml; *P* < 0.01) and slightly higher estimated glomerular filtration rate (78 vs. 73 ml/min/1.73m^2^, *P* = 0.03). Further, fewer of our patients had diabetes mellitus type 1 or 2 (13% vs. 20%; *P* = 0.04) and fewer received cardiac resynchronization therapy (50 vs. 61%; *P* = 0.01). Except for these variables, patients in the sub study and the remaining patients in the main study were similar. No significant difference was found between etiology in the two study populations, where the majority of patients had idiopathic cardiomyopathy; 78% in our sub study and 75% in the main study (Table 1). Measures of median ESV and EDV are shown in Table 2. Mean values for ESV and EDV were: ESV mean ± SD: 90 ± 65 and EDV mean ± SD: 148 ± 66. Median ESVI at rest was 35 ml/m^2^ (IQR 24 to 57) and median EDVI was 65 ml/m^2^ (IQR 52 to 86).

**Table 1:** Characteristics of study population and comparison with main study population

	Patients in PET-study (*N* = 151)	Remaining study population without COPD (*N* = 819)	*P*-value
Age (IQR) – yr.	62 (55–69)	63 (55–71)	0.33
Male sex – no. (%)	104 (69)	608 (74)	0.17
Body-mass index (IQR) – kg/m^2^	26.6 (24.1–29.8)	26.9 (23.9–30.2)	0.71
NT-pro-BNP (IQR) – pg/ml	851 (466–1848)	1220 (618–2274)	< 0.01
eGFR (IQR) – ml/min/ 1.73 m^2^	78 (63–94)	73 (58–92)	0.03
Left ventricular ejection fraction (IQR) – %	25 (20–31)	25 (20–30)	0.04
Coexisting conditions – no. (%)			
Diabetes mellitus – no. (%)	19 (13)	160 (20)	0.04
Hypertension	47 (31)	257 (31)	0.94
Left bundle branch block – no. (%)	83 (60)	480 (65)	0.22
Cause of heart failure – no. (%)			
Idiopathic	118 (78)	615 (75)	0.32
Valvular	9 (6)	31 (4)	
Hypertension	11 (7)	88 (11)	
Other	13 (9)	85 (10)	
Medications – no. (%)			
ACE-inhibitor or ARB	149 (99)	785 (96)	0.09
Beta blocker	145 (96)	747 (91)	0.05
Aldosterone receptor antagonist	86 (57)	471 (58)	0.90
Statins	60 (40)	356 (43)	0.39
Anticoagulation treatment	54 (36)	314 (38)	0.55
Acetylsalicylic acid	53 (35)	308 (38)	0.56
Device therapy – no. (%)			
CRT	75 (50)	496 (61)	0.01

*COPD* chronic obstructive pulmonary disease, *IQR* interquartile range, *NT-proBNP* N-terminal pro-brain natriuretic peptide, *eGFR* estimated glomerular filtration rate, *ACE* angiotensin-converting enzyme, *ARB* angiotensin-receptor blocker, *CRT* cardiac resynchronization therapy. Two-sided *P* value determined by Wilcoxon two-sample or chi-square test

**Table 2:** ^82^Rb-PET/CT scan data

All patients (N = 151)	
Atrial fibrillation/flutter during scan – %	30 (21)
CACS	
CACS = 0 – no. (%)	42 (28)
CACS 1–99 – no. (%)	33 (22)
CACS 100–399 – no. (%)	33 (22)
CACS 400–999 – no. (%)	22 (15)
CACS ≥1000 – no. (%)	12 (8)
Rest scan	
Rubidium-82 – Mbq	1062 (1018-1273)
Systolic blood pressure – mmHg	108 (98–118)
Diastolic blood pressure – mmHg	63 (56–69)
MAP - mmHg	77 (71–85)
Heart rate – bpm	67 (61–73)
Rate-pressure product	7137 (6840-9345)
Left ventricular ejection fraction – %	45 (34–55)
End-systolic volume – ml	71 (50–118)
End-diastolic volume – ml	134 (103–182)
Stress scan	
Rubidium-82 – Mbq	1058 (1014-1269)
Adenosine – mg	71 (63–80)
Systolic blood pressure – mmHg	109 (99–121)
Diastolic blood pressure – mmHg	62 (55–68)
MAP- mmHg	78 (71–86)
Heart rate – bpm	74 (67–80)
Rate-pressure product	7848 (6840-9345)
Left ventricular ejection fraction – %	51 (36–62)
End-systolic volume – ml	71 (45–121)
End-diastolic volume – ml	144 (119–192)

Values are medians and interquartile ranges if nothing else is indicated. *CACS* coronary artery calcium score, *MAP* mean arterial pressure

### Global myocardial blood flow and myocardial flow reserve (MFR)

**Fig. 2:**
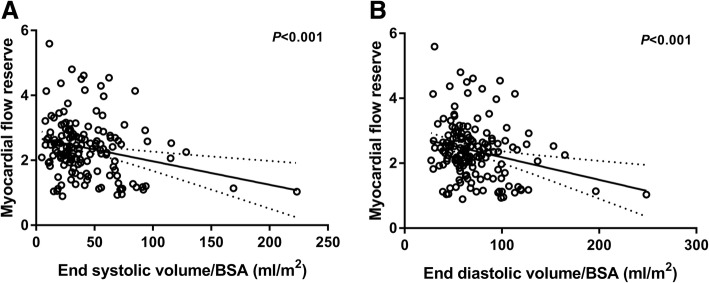
Myocardial flow reserve as a function of end-systolic volume/body surface area (**a**) and end-diastolic volume/body surface area (**b**) with 95% confidence bands of the best-fit line

Perfusion results are found in Table 3. There was a significant linear decrease in MFR both with increasing ESVI (estimate − 3.7% /10 ml/m^2^; 95% confidence interval [CI] -5.6 to − 1.8; *r* = 0.30; *P* < 0.001), (Fig. 2a) and with increasing EDVI (estimate − 3.5% /10 ml/m^2^; 95% CI -5.3 to − 1.6; *r* = 0.29; P < 0.001), (Fig. 2b).

**Table 3:** Myocardial perfusion data

All patients (N = 151)	
Median global rest MBF (IQR) – ml/g/min	0.94 (0.76–1.09)
Median global stress MBF (IQR) – ml/g/min	2.18 (1.69–2.76)
Mean MFR (95% CI)	2.38 (2.24–2.52)
Mean coronary vascular resistance rest – mmHg/(ml/g/min) (95% CI)	89 (85–93)
Mean coronary vascular resistance stress – mmHg/(ml/g/min) (95% CI)	43 (39–47)
Median TID (IQR)	1.07 (1.02–1.14)
Median SRS (IQR)	2.0 (0.0–4.0)
Median SSS (IQR)	4.0 (2.0–7.0)
Median SDS (IQR)	2.0 (1.0–5.0)

*IQR* interquartile range, *MBF* myocardial blood flow, *MFR* myocardial flow reserve, *CI* confidence interval, *TID* transient ischemic dilation, *SRS* summed rest score, *SSS* summed stress score, *SDS* summed difference score

More complex models of relation did not improve the fit. In multivariable analyses, this relationship remained significant for both variables; ESVI: estimate − 4.5% /10 ml/m^2^; 95% CI -7.9 to − 0.9; *P* = 0.02 and EDVI: estimate − 3.8% /10 ml/m^2^; 95% CI -6.8 to − 0.7; *P* = 0.02. Table 4 and Additional file 1: Table S1 show the complete results of the multivariable analyses for ESVI and EDVI respectively. In the univariable analyses, we also found a significant association between MFR and LVEF: estimate 6.1%/10%; 95% CI 1.9 to 10.5%/10%; *P* < 0.01. However, this association became non-significant in multivariable analyses both when including ESVI or EDVI and when not including volumes in the statistical model (All> 0.10 for the association between MFR and LVEF).

**Table 4:** Analyses of MFR and ESVI

Myocardial flow reserve				
	Univariable		Multivariable	
	Percent change per unit^a^ (95% CI)	*P*-value	Percent change per unit ^a^ (95% CI)	*P*-value
End-systolic volume/BSA (10 ml/m^2^)	− 3.7 (− 5.6; − 1.8)	< 0.001	−4.5 (− 7.9; − 0.9)	0.02
Male sex	−8.5 (− 20.0; 4.6)	0.19	3.9 (− 10.4; 20.5)	0.60
Age (10y)	− 8.0 (− 13.5; − 2.1)	< 0.01	−1.6 (− 8.7; 6.2)	0.68
Hypertension	0.8 (− 12.0; 15.4)	0.91	3.9 (− 9.4; 19.2)	0.58
Type 2 diabetes	−11.1 (− 24.3; 4.5)	0.15	−1.0 (− 16.1; 16.7)	0.90
Log2(NT-pro-BNP)	−3.8 (− 8.1; 0.7)	0.10	1.7 (− 3.6; 7.3)	0.54
Left ventricular bundle branch block	1.2 (− 11.3; 15.5)	0.85	5.7 (− 18.4; 9.1)	0.43
LVEF at rest (10%)	6.1 (1.9; 10.5)	< 0.01	− 4.5 (− 11.9; 3.5)	0.26
Atrial fibrillation during scan	−27.6 (− 37.7; − 16.0)	< 0.0001	−26.1 (− 38.4; − 11.4)	< 0.01
Increases in heart rate from rest to stress	1.0 (0.4; 1.5)	< 0.001	0.8 (0.0; 1.6)	0.049
CACS (100 units)	−1.6 (− 2.7; − 0.5)	< 0.01	−1.5 (− 2.6; − 0.3)	0.01

^a^Estimated differences are expressed in relative terms, i.e. as a percentage. *CI* confidence interval, *BSA* body surface area. *LVEF* Left ventricular ejection fraction. *CACS* Coronary calcium score

Median global rest MBF was 0.94 ml/g/min (interquartile range [IQR] 0.76 to 1.09) and median global stress MBF was 2.18 ml/g/min (IQR 1.69–2.76). Table 5 shows associations between rest and stress MBF and ESVI and EDVI respectively. Global stress MBF decreased significantly with increasing ESVI in both the univariable (estimate − 0.10 (ml/g/min) per (10 ml/m^2^); *P* < 0.0001) and the multivariable (estimate − 0.07 (ml/g/min) per (10 ml/m^2^); *P* = 0.048) analyses. Results at rest showed significant decrease in global rest MBF with increasing ESVI in the univariable analyses both uncorrected (estimate − 0.02 (ml/g/min) per (10 ml/m^2^); *P* = 0.01) and corrected for cardiac work (RPP) (estimate − 0.02 (ml/g/min) per (10 ml/m^2^); *P* = 0.03). No significant associations were found in the corresponding multivariable adjustment analyses. In a univariable analysis, global stress MBF also decreased with increasing EDVI (estimate − 0.10 (ml/g/min) per (10 ml/m^2^); *P* < 0.0001), but with multivariable adjustment the decrease was only borderline significant (estimate − 0.06 (ml/g/min) per (10 ml/m^2^); *P* = 0.052). We also found a significant association between global rest MBF and EDVI in the univariable analysis (estimate − 0.02 (ml/g/min) per (10 ml/m^2^); *P* = 0.03), which was not significant following multivariable adjustment (estimate 0.00 (ml/g/min) per (10 ml/m^2^); *P* = 0.99) Correcting for RPP at rest, the association between global rest MBF and EDVI were non-significant in both the univariable and multivariable analyses.

**Table 5:** Analyses of MBF and ESVI and EDVI

	Univariable		Multivariable^a^	
	MBF^b^ (95% CI)	*P*-value	MBF^b^ (95% CI)	*P*-value
Rest				
ESVI (10 ml/m^2^)	−0.02 (− 0.03 to − 0.004)	0.01	−0.003 (− 0.03 to 0.02)	0.81
EDVI (10 ml/m^2^)	−0.02 (− 0.03 to − 0.002)	0.03	0.00 (− 0.02 to 0.02)	0.99
Rest adjusted for rate-pressure product				
ESVI (10 ml/m^2^)	−0.02 (− 0.04 to − 0.002)	0.03	−0.01 (− 0.03 to 0.04)	0.73
EDVI (10 ml/m^2^)	−0.02 (− 0.03 to 0.00)	0.054	0.01 (− 0.02 to 0.03)	0.67
Stress				
ESVI (10 ml/m^2^)	−0.10 (− 0.14 to − 0.06)	< 0.0001	−0.07 (− 0.14 to − 0.001)	0.048
EDVI (10 ml/m^2^)	−0.10 (− 0.13 to − 0.06)	< 0.0001	−0.06 (− 0.12 to 0.00)	0.052

^a^All multivariable values shown are adjusted for sex, age, hypertension, diabetes, NT-pro-BNP, LV bundle branch block, LV ejection fraction, atrial fibrillation during scan, increases in heart rate from rest to stress and coronary calcium score. ^b^Change in MBF (ml/g/min). *ESVI* end-systolic volume index, *EDVI* end-diastolic volume index

### Coronary vascular resistance (CVR)

**Fig. 3:**
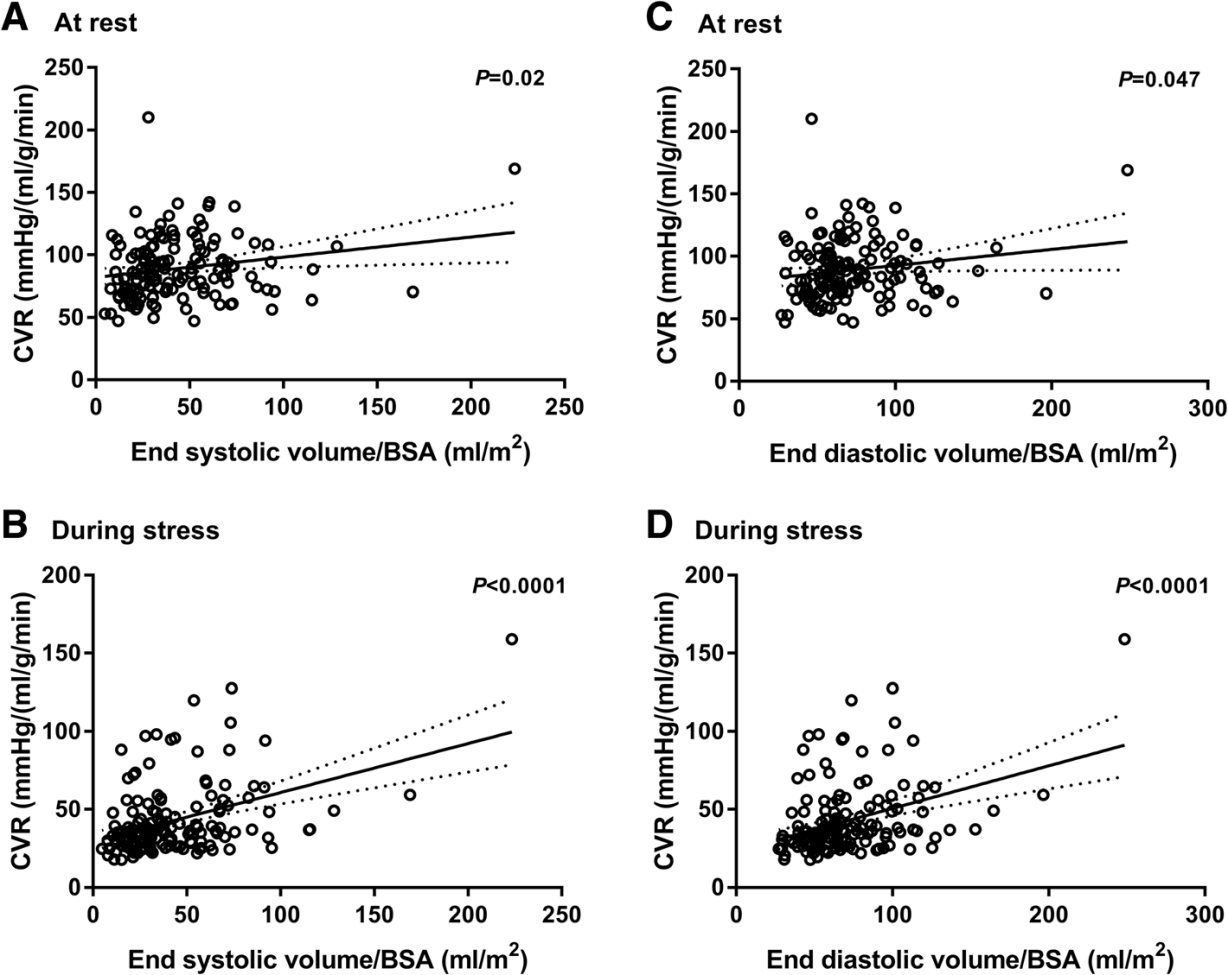
Coronary vascular resistance (CVR) as a function of end-systolic volume/body surface area at rest (**a**) and during stress (**b**) and as a function of end-diastolic volume/body surface area at rest (**c**) and during stress (**d**) with 95% confidence bands of the best-fit line

Coronary vascular resistance values at rest and during stress are also shown in Table 3. In univariable analyses, we found a significant correlation between CVR at rest and ESVI (estimate: 1.6 mmHg/(ml/g/min) per (10 ml/m^2^); 95% CI 0.3 to 2.9; *r* = 0.21; *P* = 0.02), (Fig. 3a) and between CVR at rest and EDVI (estimate: 1.3 mmHg/(ml/g/min) per (10 ml/m^2^); 95% CI 0.2 to 2.6; *r* = 0.17; *P* = 0.047), (Fig. 3c). In multivariable analyses between resting CVR and ESVI and between CVR and EDVI respectively these associations were no longer significant (*P* = 0.92 and *P* = 0.88). The statistical correlations between CVR during stress and ESVI and EDVI, respectively were strong in univariable analyses: ESVI estimate: 3.1 mmHg/(ml/g/min) per (10 ml/m^2^); 95% CI 2.0 to 4.3; *r* = 0.41; *P* < 0.0001 (Fig. 3b) and EDVI estimate: 2.7 mmHg/(ml/g/min) per (10 ml/m^2^); 95% CI 1.6 to 3.8; *r* = 0.37; *P* < 0.0001), (Fig. 3d). The associations remained significant for both variables in the multivariable analyses: ESVI estimate: 3.1 mmHg/(ml/g/min) per (10 ml/m^2^); 95% CI 1.3 to 5.0; *P* < 0.001 and EDVI estimate: 2.2 mmHg/(ml/g/min) per (10 ml/m^2^); 95% CI 0.6 to 3.8; *P* < 0.01).

### Sub analyses in patients with idiopathic cardiomyopathy

When investigating only the 118 patients with idiopathic cardiomyopathy, we found similar results for both MFR and CVR. MFR decreased with increasing ESVI (estimate − 3.5% /10 ml/m^2^; 95% CI -6.0 to − 1.1; *r* = 0.29; P < 0.01) and with increasing EDVI (estimate − 3.3% /10 ml/m^2^; 95% CI -5.7 to − 1.0; *r* = 0.28; *P* < 0.01). At stress, CVR was significantly associated with both ESVI (estimate: 3.2 mmHg/(ml/g/min) per (10 ml/m^2^); 95% CI 1.7 to 4.6; *r* = 0.42; *P* < 0.0001) and EDVI (estimate: 2.8 mmHg/(ml/g/min) per (10 ml/m^2^); 95% CI 1.3 to 4.2; *r* = 0.38; *P* < 0.001). The associations remained significant after multivariable adjustment. At rest, no significant associations were found between CVR and ESV and EDV, respectively.

### Myocardial perfusion defects

Summed rest-, stress- and difference scores (SRS, SSS and SDS) were assessed in all patients (Table 3). We found that the median SSS was in the category of mildly abnormal (4.0; IQR 2.0 to 7.0) corresponding to 5–10% of the myocardium with perfusion abnormalities. In both univariable and multivariable analyses, SSS and SRS were significantly higher with increasing ESVI (SSS: estimate 0.9 per (10 ml/m^2^), 95% CI 0.7 to 1.3, *P* < 0.0001 and SRS: estimate 0.9 per (10 ml/m^2^), 95% CI 0.7 to 1.1, *P* < 0.001) and multivariable (SSS: estimate 0.9 per (10 ml/m^2^), 95% CI 0.3 to 1.4, *P* < 0.01 and SRS: estimate 0.8 per (10 ml/m^2^), 95% CI 0.3 to 1.2, *P* < 0.001). No significant association was found between ESVI and SDS either in a univariable or a multivariable analysis. Results were similar for EDVI. Association with SSS and SRS were significant univariable (SSS: estimate 0.9 per (10 ml/m^2^), 95% CI 0.6 to 1.2, *P* < 0.0001 and SRS: estimate 0.8 per (10 ml/m^2^), 95% CI 0.6 to 1.0, *P* < 0.001), whereas there were no significant relation between SDS and EDVI (SDS: estimate 0.1 per (10 ml/m^2^), 95% CI -0.04 to 0.3, *P* = 0.14). In multivariable analyses the association between EDVI and SSS as well as SRS remained significant (SSS: estimate 0.6 per (10 ml/m^2^), 95% CI 0.1 to 1.1, *P* = 0.02 and SRS: estimate 0.6 per (10 ml/m^2^), 95% CI 0.2 to 1.0, *P* < 0.01). Results for SSS and SRS remained significant associated with both ESVI and EDVI in the univariable analyses, when only including the 88 patients with more than 5% abnormal perfusion (SSS ≥4).

## Discussion

This study of MFR assessed by ^82^Rb-PET/CT in patients with non-ischemic systolic heart failure showed that MFR decreased linearly with increases in ESVI and EDVI. These findings remained significant when adjusting for relevant covariates. Further, we found a positive association between stress CVR and ESVI as well as stress CVR and EDVI. Of our patients 64% or 46% had resting ESV higher than 59 ml or 78 ml and 72% or 66% had resting EDV higher than 108 ml or 115 ml. These values corresponded to one standard deviation above the mean normal values in the background literature.

Our results concur with previous findings of decreased MFR in patients with non-ischemic systolic heart failure [4–6, 24], and with findings of correlation between impaired myocardial perfusion and increases in ESV and EDV in patients with dilated cardiomyopathy [12–14]. The current study adds knowledge about the associations between MFR from ^82^Rb-PET/CT, as gold standard quantitative measurement of myocardial perfusion, and ESVI and EDVI in patients with varying types of non-ischemic systolic heart failure. In these patients, we found a linear association between increasing ESVI and EDVI and decreasing MFR. These findings have, to our knowledge, not previously been explored in patients with non-ischemic systolic heart failure using ^82^Rb-PET/CT. Previous studies have investigated patients with dilated cardiomyopathy using either echocardiography with coronary Doppler catheter to measure coronary flow reserve [12, 13] or SPECT with technetium-99 m methoxyisobutylisonitrile (^99m^Tc-MIBI) washout rate as a surrogate measure [14] which are both inferior to PET in measuring myocardial perfusion.

The linear relationship between increasing ESVI and EDVI and the decreasing MFR may suggest an association between degree of dilatation and microvascular disease progression. Studies on dilated cardiomyopathy have not found any correlation between myocardial perfusion abnormalities and extent of myocardial fibrosis [25, 26] indicating that more complex mechanisms determine the myocardial perfusion.

Several experimental observations about myocardial perfusion have previously been made. Tsagalou et al. found that in patients with dilated cardiomyopathy, decreased MFR obtained by thermodilution was associated with decreased myocardial capillary density measured in endomyocardial biopsies. These results support that angiogenic therapies with intramyocardial delivery of different kinds of vascular endothelial growth factors could be considered for future treatment of heart failure [24]. In contrast, a review article by Linzbach et al. described eccentric cardiomyopathy as a structural dilation with a rearrangement of muscle fibers and sufficient capillary supply but a retarded growth of the coronary arteries and coronary ostia with more frequent atherosclerotic lesions [27]. Another explanation may be a myogenic response from coronary arterioles related to oxygen consumption [6, 28].

In the current study we found a significant positive association between CVR during stress and ESVI and EDVI that remained significant in a multivariable analysis. One explanation could be that the lower MFR is simply due to a higher CVR at stress in these patients, perhaps caused by higher myocardial tissue pressure in diastole [29]. Median global rest MBF was within the normal range, although in the high end, while stress MBF on the other hand was slightly below the normal range. In addition, a decrease in stress MBF was associated with increasing ESVI univariable and multivariable and increasing EDVI in a univariable analysis (although this association became borderline significant in a multivariable analysis). This suggests that the correlation between impaired MFR with increasing ESVI and EDVI may primarily be explained by decreased stress MBF and increased CVR during stress.

It is well known that impaired MFR is associated with increased CACS both in patients with intermediate risk of coronary artery disease and in asymptomatic adults [30, 31]. Also in our population we found a significant association between MFR and CACS in the multivariable analyses. However, this association did not change the associations between MFR and ESVI and EDVI, indicating LV volume indexes are independently associated with impaired perfusion in patients with non-ischemic systolic heart failure.

It remains uncertain whether impaired myocardial perfusion causes dilatation or dilatation leads to impaired perfusion. If the first scenario is true, treatment focused on the impaired perfusion may prevent further dilatation. To investigate the second scenario further, it would be interesting to measure MFR before patients initiate treatment for heart failure and when they are fully up titrated to observe if MFR improves with reversibility in dilatation.

### Limitations

This study was conducted in a group of patients with non-ischemic systolic heart failure from a larger randomized clinical trial [15]. The patients in our study population may have been healthier than the total study population of the main study (lower NT-proBNP, higher eGFR and fewer with diabetes mellitus). This could possibly be caused by death or exclusion of the sickest patients before the sub study inclusion started leaving a healthier population for inclusion to ^82^Rb-PET/CT scan. We adjusted for various potential risk factors in the multivariable adjustment and the association between MFR and ESVI as well as between MFR and EDVI stayed significant. We chose not to include LV mass in the multivariable adjustment, because of a very strong correlation between ESVI and LV mass (*r* = 0.92) and EDVI and LV mass (*r* = 0.90), respectively. However, if LV mass was included in the model, the association between both MFR and ESVI and MFR and EDVI remained significant, while LV mass was not independently correlated with MFR. Patients could be included in our study with some extent of coronary arteriosclerosis. However, this was not the cause of their heart failure and CACS did not explain the association between MFR and ESVI and EDVI, respectively. Additionally, at the time of 82Rb-PET/CT, no patients complained of angina, none had received interventional treatment and only 2.6% of patients received calcium antagonists and 2.0% received long lasting nitrates.

## Conclusion

In patients with non-ischemic systolic heart failure, impaired MFR was significantly associated with increased left ventricular ESV and EDV. This is the first study using ^82^Rb-PET/CT with a quantitative measure of myocardial perfusion to show a linear association between myocardial perfusion and end-systolic- and end-diastolic volumes in patients with non-ischemic systolic heart failure. Whether microvascular dysfunction plays a role in heart failure development remains uncertain. Our results imply that myocardial perfusion measured as MFR may be a useful tool to elucidate this. Clinical trials investigating changes in MFR and its relation to treatment responses may clarify the clinical use of MFR in patients with non-ischemic systolic heart failure.

## Additional file

Additional file 1: **Table S1**. Multivariable analysis with myocardial flow reserve as the dependent variable including end-diastolic volume index, sex, age, hypertension, diabetes, NT-pro-BNP, LV bundle branch block, LV ejection fraction, atrial fibrillation during scan, increases in heart rate from rest to stress and coronary calcium score as possible explanatory variables. (DOCX 19 kb)

## Abbreviations

^82^Rb-PET/CT: Rubidium-82 positron emission tomography / computed tomography
CACS: Coronary artery calcium score
CRT: Cardiac resynchronization therapy
CVR: Coronary vascular resistance
EDVI: End-diastolic volume index
ESVI: End-systolic volume index
MBF: Myocardial blood flow
MFR: Myocardial flow reserve
MPI: Myocardial perfusion imaging
RPP: Rate pressure product
SDS: Summed difference score
SRS: Summed rest score
SSS: Summed stress score
