# Supplementary material for: Impaired myocardial perfusion is associated with increasing end-systolic- and end-diastolic volumes in patients with non-ischemic systolic heart failure: a cross-sectional study using Rubidium-82 PET/CT
Source: BMC Cardiovasc Disord. 2019 Mar 22;19:68. doi: 10.1186/s12872-019-1047-x (PMC6431039; doi:10.1186/s12872-019-1047-x)
Supplement: Supplementary file 1 — Table S1. Multivariable analysis with myocardial flow reserve as the dependent variable including end-diastolic volume index, sex, age, hypertension, diabetes, NT-pro-BNP, LV bundle branch block, LV ejection fraction, atrial fibrillation during scan, increases in heart rate from rest to stress and coronary calcium score as possible explanatory variables. (DOCX 19 kb) [file 12872_2019_1047_MOESM1_ESM.docx]

**Supplementary table S1. Analyses of MFR and EDVI**

| **Myocardial flow reserve** | | | | |
| --- | --- | --- | --- | --- |
|  | **Univariable** | | **Multivariable** | |
|  | **Percent change per unit* (95 % CI)** | ***P*-value** | **Percent change per unit* (95 % CI)** | ***P*-value** |
| End-diastolic volume/BSA (10ml/m^2^) | -3.5 (-5.3 ; -1.6) | <0.001 | -3.8 (-6.8 ; -0.7) | 0.02 |
| Male sex | -8.5 (-20.0; 4.6) | 0.19 | 4.3 (-10.0 ; 21.0) | 0.57 |
| Age (10y) | -8.0 (-13.5; -2.1) | <0.01 | -1.7 (-8.9 ; 6.0) | 0.66 |
| Hypertension | 0.8 (-12.0; 15.4) | 0.91 | 4.4 (-9.1 ; 19.7) | 0.54 |
| Type 2 diabetes | -11.1 (-24.3; 4.5) | 0.15 | -1.6 (-16.5 ; 16.0) | 0.85 |
| Log2(NT-pro-BNP) | -3.8 (-8.1; 0.7) | 0.10 | 1.6 (-3.7 ; 7.2) | 0.55 |
| Left ventricular bundle branch block | 1.2 (-11.3; 15.5) | 0.85 | 6.8 (-19.3 ; 7.7) | 0.34 |
| LVEF at rest (10 %) | 6.1 (1.9 ; 10.5) | <0.01 | -3.0 (-9.7 ; 4.1) | 0.39 |
| Atrial fibrillation during scan | -27.6 (-37.7; -16.0) | <0.0001 | -27.4 (-39.5 ; -12.9) | <0.001 |
| Increase in heart rate from rest to stress | 1.0 (0.4 ; 1.5) | <0.001 | 0.8 (0.0 ; 1.6) | 0.06 |
| CACS (100 units) | -1.6 (-2.7 ; -0.5) | <0.01 | -1.4 (-2.6 ; -0.3) | 0.02 |

*Estimated differences are expressed in relative terms, i.e. as a percentage. CI confidence interval. BSA body surface area. LVEF Left ventricular ejection fraction. CACS Coronary calcium score.
